# Lyoprotection and stabilization of laccase extract from *Coriolus hirsutus*, using selected additives

**DOI:** 10.1186/s13568-018-0683-3

**Published:** 2018-09-27

**Authors:** Christelle Bou-Mitri, Selim Kermasha

**Affiliations:** 1 Faculty of Nursing and Health Sciences, Notre Dame University Zouk Mosbeh, P.O.Box: 72, Zouk Mikael, Lebanon; 2 Department of Food Science and Agricultural Chemistry, McGill University, 21,111 Lakeshore, Ste-Anne de Bellevue, QC H9X 3V9 Canada

**Keywords:** Laccase, *Coriolus hirsutus*, Stabilization, Lyoprotectants, Additives

## Abstract

The development of stable lyophilized laccase, obtained from *Coriolus hirsutus*, using a wide range of temperature treatments and storage conditions, was investigated. Using selected lyoprotectants, including, dextran 6 kDa, sucrose and a mixture of sodium benzoate, potassium sorbate and sorbitol (BKSS) (1.5:1.0:98.5; w/w/w) resulted by 2.4, 1.4 and 1.8-fold increase in laccase activity after lyophilization as compared to the fresh enzyme, respectively, whereas the addition of mannitol preserved 98.2% of its activity. Using 2.5% (w/v) dextran (15–25 kDa) or mannitol appeared to be the most appropriate lyoprotectants for the laccase activity. The laccase stability of the lyophilized enzymatic extract was greatly enhanced with the presence of mannitol, with 96.2, 38.9 and 24.7% of residual activity after 4 weeks of storage at − 80, 4 and 25 °C, respectively. The inactivation constant (*k*_inactivation_) value and the amount required to decrease 50% of the laccase activity (*C*_1/2_) showed that Carbowax^®^ polyethylene glycol (PEG)-8000 was the most appropriate additive for laccase activity, followed by glycerol and CuSO_4_. When the enzymatic extract was incubated at 50 °C in the presence of either CuSO_4_, PEG-8000 or glycerol, the time required to decrease 50% of the laccase initial activity (*t*_50_), were 52.9, 54.6, 50.2 h, respectively, as compared to that of the control trial of 38.9 h.

## Introduction

Laccases (EC 1.10.3.2) are *o*- and *p*- diphenol: dioxygen-oxidoreductase (Dalfard et al. 2006). Laccases belong to the polyphenol oxidases that catalyze the oxidation of a large number of phenolic and non-phenolic compounds, with a parallel reduction of the molecular oxygen as the electron acceptor (Tarasi et al. 2018).

Laccases are widespread in nature and they are produced by plants, fungi, bacteria, insects and crustacean. Most laccases have been isolated from white rot basidiomycetes, since mostly fungal laccases were found as extra-cellular enzymes (Strong and Claus 2011).

The broad substrate specificity, the high catalytic rate and the ability of laccases to use the environmental oxygen as a co-factor, made them highly useful as biocatalysts in versatile biotechnological applications, including lignin biodegradation, biobleaching, detoxification, organic synthesis, biosensing and biografting (Freire et al. 2001; Gianfreda and Bollag 2002; Jeon and Chang 2013; Thakur et al. 2016; Tarasi et al. 2018; Yesilada et al. 2018; Agrawal et al. 2019; Rocha-Martín et al. 2018). As green catalysts, laccases have also been increasingly employed in various industrial, environmental and medical applications leading to rapid growth in the demand for these enzymes (Liu et al. 2016). Gap between production and demand was reported owing to their high production and purification cost as well as to their poor stability (Nunes and Kunamneni 2018).

Lyophilization, thermal treatments, reaction process, protease as well as laccase-catalyzed polymerization, depolymerization and detoxification reaction products could destabilize the enzyme (Chansanroj and Thanawattanawanich 2016). Various chemicals were employed to stabilize laccase. The results have shown that the enzyme activity and stability was enhanced in presence of polyethylene glycol (PEG) (Kim and Nicell 2006a, b; Modaressi et al. 2005; Ghosh et al. 2008), CuSO_4_ (Baldrian and Gabriel 2002; Papinutti et al. 2008) mannitol and glycerol (Papinutti et al. 2008). The use of polysaccharides, including guar gum, starch, agarose and agar, for the prevention of the laccase denaturation and retention of the enzyme activity for long period in solution state, was also reported (Poonkuzhali and Palvannan 2011; Poonkuzhali et al. 2011). Several additives were also employed to enhance the stability of the vacuum dried laccase including polyvinyl alcohol, dextran, lactitol and polyacrylic (Stepanova et al. 2003).

Although the literature reported on the stability of laccase in relation to the vacuum drying (Stepanova et al. 2003), spray drying (Liu et al. 2016), thermal treatment (Papinutti et al. 2008), storage and biocatalysis (Kim and Nicell, 2006a, b; Papinutti et al. 2008); there is a wide range of discrepancy in the reported experimental findings especially due to the source, the nature of the enzyme and its application. Moreover, to our knowledge there is lack of information regarding the production of stable lyophilized laccase with high activity and extended shelf life.

The present work is part of ongoing research in our laboratory aimed at the biotechnological applications of laccase in non-conventional media. The aim of the present study was to prepare stable lyophilized powdered laccase with long-term conservation of its activity and high operating stability. The stabilization of laccase activity of the enzymatic extract, obtained from *Coriolus hirsutus*, after lyophilization using selected additives, was investigated. It was aimed to use the enzymatic extract prior purification to avoid further increase of the cost of process. The efficiency of selected lyoprotectants on laccase thermal and storage stability as well as the effect of selected additives on laccase activity and stability in the reaction media were also evaluated.

## Materials and methods

### Materials

*Coriolus hirsutus* (MYA-828) was obtained from the American Type Culture Collection (ATCC; Manassas, VA). The additives include KCl and mannitol (BDH Inc.; Toronto, ON), glycerol (MP Biomedicals; Solon, OH), sucrose and CuSO_4_ (ACP Chemical Inc.; Montreal, QC) as well as bovine serum albumin (BSA), dextran, sodium benzoate, sorbitol, potassium sorbate and the substrate syringaldazine (SG) (Sigma Chemical Co.; St. Louis, MO). The anhydrous ethanol was obtained from Commercial Alcohols Inc. (Branchville, QC). Other chemicals used for the preparation of reagents and buffers were purchased from Fisher Scientific (Fair Lawn, N.J.) and prepared in deionized water, using Milli Q plus (Millipore; Milford, MA).

### Preparation of the enzymatic extract

As indicated by the ATTC protocol, *C. hirsutus* was maintained through periodic transfer onto malt agar media plates and incubated at 20 °C. For laccase production, a basal medium was prepared using the method described by Shin and Lee (2000). The fermentation of *C. hirsutus* as well as the recovery and enrichment of the enzymatic extract were carried out according to Taqi (2012). The production of laccase was induced with the addition of 50 mL ethanol/L of culture medium. After 14 days of incubation, the mycelium pellets were removed with cheese cloth and the extracellular enzyme recovered from the culture media was ultra-filtered through a Prep/Scale TFF Cartridge (2.5 ft^2^) (Millipore) of polyethersulfone low protein-binding membranes of 10 kDa cut-off filter and a pressure of 10 psi. All steps were performed at 4 °C unless otherwise stated.

### Protein determination

The protein content of the enzymatic extracts was determined according to a modification of Lowry method (Hartree 1972), using BSA (Sigma Chemical Co.) as a standard for the calibration curve.

### Enzyme assay

Laccase activity was performed following the optimized procedure described by Taqi (2012). Lyophilized laccase extract was suspended in sodium acetate buffer (0.1 M, pH 5.0). A 0.2 mL aliquot of enzyme suspension or the fresh enzyme extract (0.2–0.5 mg protein/mL) was added to a total 0.7 mL of the acetate buffer, containing 15 µL of 4.0 mM SG solution in ethanol. Laccase activity was assayed by measuring the initial oxidation rate of SG monitored at 525 nm (ε_525_, 65,000 cm^−1^·M^−1^), using DU 650 spectrophotometer (Beckman Instruments Inc.; San Raman, CA). One unit of laccase activity was defined as 1 mol of product per mL of enzyme per min. The enzymatic reaction was performed at 55 °C, otherwise mentioned. All laccase assays were performed in triplicate and run in tandem with control trials containing 15 µL of 4.0 mM SG, but without an enzyme extract, in a total volume of 0.7 mL.

### Effect of selected lyoprotectants on laccase stability

Prior to lyophilization, selected lyoprotectants (w/v), including 0.5% BSA, 5% mannitol, 5% sucrose, 1% dextran 6 kDa, 70% (w/w) KCl or a 5% of the mixture of sodium benzoate, potassium sorbate and sorbitol (BKSS) (1.5:1.0:98.5; w/w/w), were added to the fresh ultra-filtered concentrated *exo*-laccase. The effect of dextran, with a molecular weight of 1.5, 6, 15–25, 40 and 70 kDa, as well as mannitol with a wide range of concentration (0–10%, w/v) on the stability of laccase was investigated and compared to the lyophilized enzyme without additive (control). After lyophilization, the enzymatic preparation was reconstituted in the acetate buffer and the laccase activity was assayed (Hall et al. 2008).

### Effect of selected lyoprotectants on laccase thermostability

The effect of temperature on the laccase stability, prepared with 2.5% (w/v) dextran or mannitol, was investigated. The lyophilized laccase preparations were reconstituted in the acetate buffer and pre-incubated for 2 h in a wide range of temperatures, 4, 20, 30, 40, 45, 50, 55, 60 and 70 °C. In addition, the different laccase preparations were reconstituted in the acetate buffer and incubated in water-bath for 0–24 h at 4, 25 and 50 °C. The treated enzymatic extracts were immediately cooled down with the use of an ice-bath before measuring the laccase activity, using the standard assay.

### Effect of selected lyoprotectants on laccase storage stability

The lyophilized enzymatic preparations, including that prepared with 2.5% (w/v) mannitol as well as with dextran 15–25 kDa, were stored at 4, 25 and − 80 °C for a period of 0–4 weeks. The laccase activity was determined each week, using the standard assay.

### Effect of selected additives on laccase activity

The effect of selected additives, including CuSO_4_, Carbowax^®^ PEG-8000 and glycerol, on laccase activity of the enzyme prepared with 2.5% (w/v) mannitol was investigated, by varying their concentrations in the reaction mixture from 0 to 10% (w/v), according to the procedure described by Hall et al. (2008). The laccase relative specific activity was calculated and defined as the concentration of oxidized syringaldazine (nmol product/mg protein/min) in the reaction trial containing the optimum additive concentration relative to that without additive. All trials were performed in triplicate and run in tandem with the control without additive. Relative percentage standard deviation was defined as the standard deviation of laccase triplicate trial divided by their respective means, multiplied by 100.

The inactivation rate of laccase activity was estimated by calculating the first-order inactivation constant (*k*_inactivation_) on semi logarithm plots. The amount (%, w/v) of the additive, required to decrease half of the initial laccase activity (C_1/2_), was calculated by using the linear equation:

1 $$\ln \;(\text{A}/\text{A}_{0} ) = - k_{\text{inactivation}} \text{C}$$

where, C is the concentration of the chemical additive; *A* and *A*_*0*_ are the laccase activities at C concentration of the chemical additive and without it, respectively.

The rate constant of inactivation (*k*_inactivation_), expressed as 1/% (w/v) of the additive per reaction volume, was estimated by plotting the ln percentage residual laccase activity versus the percentage of additive in the reaction trial for laccase. The C_1/2_, expressed in % (w/v) of the additive per reaction volume, defined as the additive concentration at which 50% of the initial activity was reached.

### Effect of selected additives on laccase thermostability

The effect of selected additives, including CuSO_4_, Carbowax^®^ PEG-8000 and glycerol, on laccase thermostability at 4, 25, 37 and 50 °C, was evaluated by pre-incubating the reconstituted lyophilized enzymatic extract. Samples were withdrawn after 7 days of incubation. The residual laccase activity was measured, using the standard assay. Residual laccase activity (%) was calculated by dividing the specific activity (nmol product/mg protein/min) of a given sample after 168 h of incubation at a defined temperature to that at time 0, multiplied by 100.

The thermal deactivation rate of laccase, at 50 °C, in presence of the selected chemical additives, was estimated by calculating the first-order deactivation constant (*k*_t_) on semi logarithm plots. Samples were withdrawn at different incubation times. The residual laccase activity was measured, using the standard assay. The thermal stability time (h) required decreasing 50% of the initial laccase activity (*t*_50_) was calculated using the equation:

2 $$t_{50} = \ln \;(2)/k_{\text{t}}$$

### Statistical analysis

Data were expressed as means of triplicate trials and their respective standard deviation (SD). The percent relative standard deviation (RSD) was calculated as the SD divided by the mean multiplied by 100. Correlation analyses were performed, using the SigmaPlot-Systat Software V. 11 (Systat Software Inc., Chicago, IL). Data were analyzed, using PROC ANOVA analysis, performed with the same statistical program. A post hoc comparison was made, using Tukey’s test. Values of *P *< 0.05 were considered to be significant.

## Results

### Effects of selected lyoprotectants on laccase stability

The effects of selected lyoprotectants, including BSA, mannitol, sucrose, dextran 6 kDa, KCl and the mixture BKSS, were investigated in terms of their efficiency to maintain the laccase activity during the lyophilization of the enzymatic extract and to enhance its solubility. Although Table 1 shows that the residual laccase activity of the lyophilized enzymatic extract of the control trial was 92.1%, the solid protein was not easily reconstituted or manipulated. The addition of 70% (w/w) of KCl inhibited completely the laccase activity, whereas the presence of 0.5% (w/v) BSA resulted by 17.3% of the residual enzyme activity. The results also show that the addition of 5% (w/v) mannitol maintained 98.2% of the original laccase activity. In addition, the laccase activity of the lyophilized enzymatic extract was determined to be 2.4, 1.4 and 1.8-fold higher than that of the fresh one, in the presence of 1% dextran 6 kDa, 5% sucrose and 5% of the mixture BKSS, respectively. The addition of BSA, mannitol, dextran 6 kDa and KCl resulted by a higher solubility of the enzymatic extract, whereas the use of sucrose and the mixture BKSS resulted in a collapsed crystalline formulation of poor solubility.

**Table 1:** Effect of different lyoprotectants on laccase stability, nature of the extract and its solubility

Extract	Residual laccase activity (%)^a^	Nature of extract^b^	Solubility^c^
Fresh without additive	100.0	Liquid	n.d.
Lyophilized without additive	92.1 (6.2)^e^	Sticky	+
Lyophilized with 0.5% (w/v) BSA	17.3 (11.6)	Powder	+++
Lyophilized with 5% (w/v) mannitol	98.2 (2.3)	Powder	+++
Lyophilized with 1% (w/v) dextran 6 kDa	241.0 (5.4)	Powder	+++
Lyophilized with 5% (w/v) sucrose	141.2 (14.0)	Collapsed	−
Lyophilized with 5% (w/v) mixture (BSKS)^d^	184.3 (2.5)	Collapsed	++
Lyophilized with 70% (w/w) KCl	n.d.	Powder	+++

*n.d.* Not detected

^a^Residual laccase activity (%) was defined as the specific activity (nmol product/mg protein/min) of the lyophilized trial in comparison to that of the fresh ultrafiltrated enzymatic extract before lyophilization without additive

^b^Nature of the extract was defined as the final texture of the lyophilized enzymatic

^c^Degree of solubility of the enzyme preparation in sodium acetate buffer (0.1 M, pH 5.0), after lyophilization, was expressed with qualitative evaluation +++, ++, + and − from the highest solubility to the lowest one, respectively, taking into consideration the required time for solubilization

^d^Mixture was composed of sodium benzoate/potassium sorbate/sorbitol (BKSS) (1.5:1.0:98.5, w/w/w)

^e^Relative percentage standard deviation was defined as the standard deviation of laccase triplicate trial divided by their respective means, multiplied by 100

### Effect of dextran molecular weight on laccase stability

**Fig. 1:**
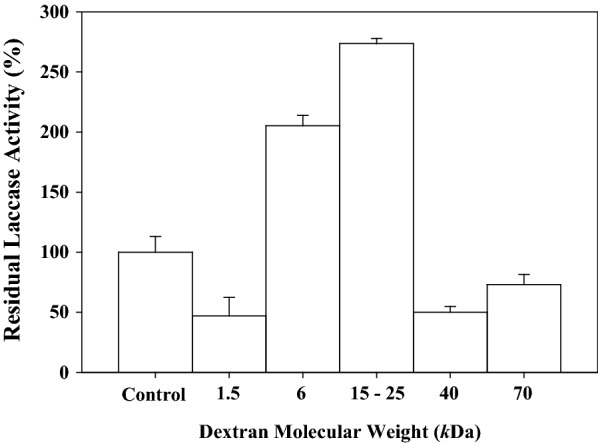
Effect of dextran molecular weight on the residual laccase activity after lyophilization, using syringaldazine as substrate. The residual laccase activity was defined as the specific activity of lyophilized enzyme to that of the lyophilized enzyme trial that is lacking the additive (Control)

The effect of dextran molecular weight (1.5, 6, 15–25, 40 and 70 kDa) on the laccase stability of the lyophilized enzymatic extract was investigated. The results (Fig. 1) show that the use of dextrans 15–25 or 6 kDa resulted in a significant increase in laccase activity by 2.6 and twofold, respectively, as compared to the lyophilized extract trial without additive. However, using dextrans 1.5, 40 and 70 kDa resulted in a residual laccase activity of 47.0, 50.1 and 73.1%, respectively.

### Effects of dextran and mannitol concentrations on laccase stability

**Fig. 2:**
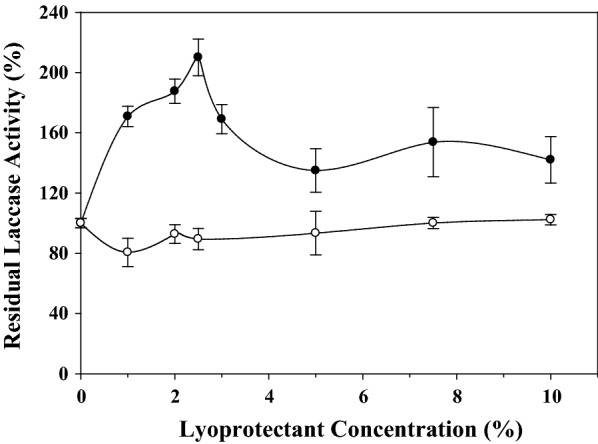
Effect of the lyoprotectant concentration (%, w/v) of dextran 5 to 25 kDa (black circle) and mannitol (white circle) on the residual laccase activity. The residual laccase activity was defined as the specific activity of lyophilized enzyme to that of the lyophilized enzyme trial that is lacking the additive

The effects of dextran (15–25 kDa) and mannitol concentrations were investigated in term of their efficiency to stabilize the laccase activity during lyophilization. Figure 2 indicates that the addition of 2.5% (w/v) dextran (15–25 kDa) resulted by a 2.1-fold activation of laccase activity; whereas it was increased from 80 to 102%, with the increase in mannitol concentration, from 1 to 10%.

### Thermostability of laccase

#### Effect of temperature on laccase stability

**Fig. 3:**
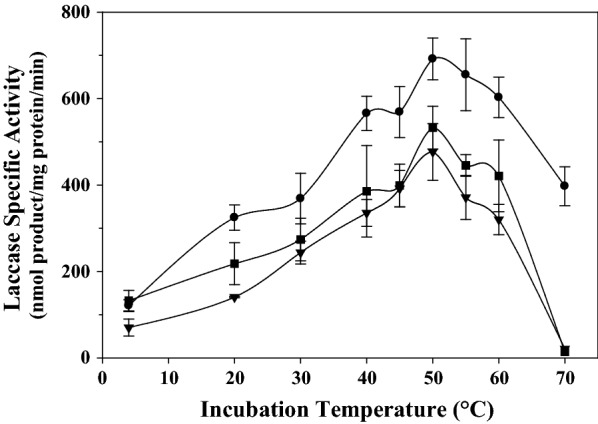
Thermostability profile of the lyophilized laccase activity, after 2 h of incubation at different temperatures, assayed with syringaldazine as substrate. The reaction mixture contains the enzymatic preparations including the control being the lyophilized enzyme without additive (black circle) with 2.5% (w/v) mannitol (black down-pointing triangle) and 2.5% (w/v) dextran 15 to 25 kDa (black square) as additive

The effect of lyoprotectants on laccase thermostability was investigated. The results (Fig. 3) show that the different laccase preparations, including the control trial and those treated with dextran and with mannitol demonstrated similar thermostability profiles, where the enzyme retained its full activity after 2 h of incubation up to 50 °C.

The energy required for the maximum activation of laccase of the enzymatic preparations, without any additive, as well as those with dextran or with mannitol, were of 26.9, 31.2 and 21.5 kJ/mol, with a coefficient Q_10_ of 1.4, 1.4 and 1.5, respectively (Data Not Shown). These findings suggest that the activity of laccase preparations using mannitol as lyoprotectant was the most prone to variation with the increase of temperature as compared to that with dextran and the control.

#### Effect of incubation time on laccase stability

**Fig. 4:**
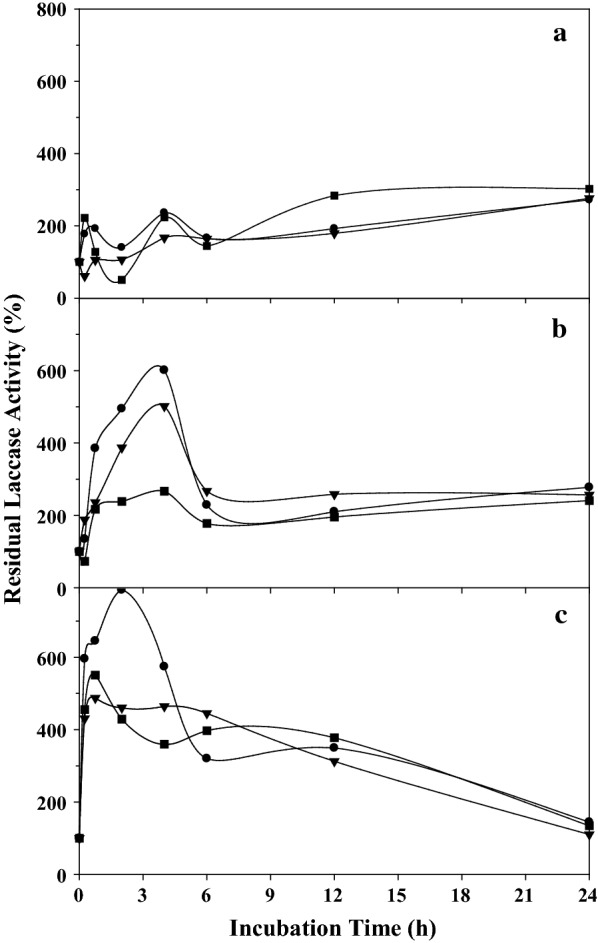
The thermostability profiles of the residual laccase activity in the lyophilized enzymatic extract from *Coriolus hirsutus*, after incubation at **a** 4, **b** 25 and **c** 50 °C. The reaction mixture contains the enzymatic preparations including the control being the lyophilized enzyme without additive (black circle) with 2.5% (w/v) mannitol (black down-pointing triangle) and 2.5% (w/v) dextran 15 to 25 kDa (black square) as additive. The residual laccase activity (%) was defined as the specific activity of each treated enzyme extract at a defined temperature and time in comparison to that at time zero of each incubation temperature

The thermostability of laccase activity of the enzyme preparations, including the control trial and those treated with dextran and with mannitol, was investigated by incubating the reconstituted enzyme preparation in the acetate buffer at different temperatures and incubation times. The thermostability profiles (Fig. 4) show that the residual laccase activity, after 24 h of incubation at 4, 25 and 50 °C, for the control trial was 271, 277 and 98%, respectively; it was also 302, 240 and 78% with 2.5% (w/v) dextran (15 to 25 kDa) and 276, 256 and 89% with 2.5% (w/v) mannitol, respectively. The results also showed high thermostability of the laccase prepared with mannitol and the control when incubated at 25 and 50 °C for 1–4 h as compared to shorter incubation time.

#### Laccase storage stability

The stability of the lyophilized laccase extracts, prepared in the presence of 2.5% (w/v) dextran or mannitol and stored for different periods and temperatures, was investigated. The results (Table 2) indicate that mannitol was the most effective stabilizer, with 96.2, 38.9 and 24.7% of residual laccase activity after 4 weeks of storage at − 80, 4 and 25 °C, respectively; whereas in the absence of any additive, it decreased steadily to 56.6, 8.5 and 17.8% at − 80, 4 and 25 °C, respectively. In the presence of dextran, it decreased to 57.1, 19.8 and 0% after 1 week of storage at − 80, 4 and 25 °C, respectively.

**Table 2:** Long-term stability of laccase activity of the lyophilized enzymatic extract from *Coriolus hirsutus*, prepared with 2.5% (w/v) mannitol or dextran 15–25 kDa

Storage (week)	− 80 °C			4 °C			25 °C		
	Control^b^	Mannitol	Dextran	Control^b^	Mannitol	Dextran	Control^b^	Mannitol	Dextran
Relative residual laccase activity (%)^a^									
0	100.0	100.0	100.0	100.0	100.0	100.0	100.0	100.0	100.0
1	85.0	96.9	57.1	48.1	95.3	19.8	46.3	49.7	0.0
2	78.6	95.8	50.6	28.2	28.3	21.0	24.4	25.6	0.0
3	78.5	101.9	47.0	8.8	33.9	9.0	16.4	26.0	0.0
4	56.6	96.2	38.5	8.5	38.9	4.4	17.8	24.7	0.0

^a^Relative residual laccase activity (%) was calculated by dividing the specific activity (nmol product/mg protein/min) of a given sample at defined storage time and temperature to that at time 0, multiplied by 100. The enzyme activity was assayed with syringaldazine as substrate

^b^Control was defined as the lyophilized enzyme without additive

#### Effects of concentrations of selected additive on laccase activity

Table 3 summarizes the experimental findings for the effects of selected additives, including CuSO_4_, PEG-8000 and glycerol, on laccase activity. The addition of 0.02% (w/v) PEG-8000 or 0.4% (w/v) glycerol to the reaction medium showed an increase of 10% in the laccase activity, whereas the addition of 0.002% (w/v) CuSO_4_ resulted by a 13% decrease of the enzyme activity.

**Table 3:** The effect of selected additives used in the reaction mixture on the laccase specific activity of an enzymatic extract from *Coriolus hirsutus*

Additive	Relative concentration (%)^a^	Relative specific activity (%)^b^
None	–	100
CuSO4	0.002	87.7 (7.0)^c^
PEG-8000	0.02	117.2 (6.9)
Glycerol	0.4	118.2 (9.4)

^a^Relative concentration of the additive was expressed in percent (w/v), was the one at which the laccase specific activity was at its optimum

^b^Relative specific activity was calculated and defined as the concentration of oxidized syringaldazine (nmol product/mg protein/min) in the reaction trial containing the optimum additive concentration relative to that without additive

^c^Relative percentage standard deviation was defined as the standard deviation of laccase triplicate trial divided by their respective means, multiplied by 100

The effect of additive concentrations on the laccase activation rate was also investigated. The results (Table 4) show that PEG-8000 was the most appropriate additive for laccase activity, followed by glycerol, with an inactivation rate constant (*k*_inactivation_) of 0.088 and 0.103, respectively. C_1/2_, which is the concentration (%, w/v) of PEG-8000, glycerol and CuSO_4_ required to decrease 50% of the initial laccase activity, were 7.8, 6.7 and 1.6% (w/v), respectively.

**Table 4:** The effect of selected additives used in the reaction mixture on laccase activity of an enzymatic extract, obtained from *Coriolus hirsutus* in aqueous medium composed of acetate buffer (0.1 M, pH 5.0), using syringaldazine as substrate

Additive inactivation parameter	Additive		
	CuSO_4_	PEG-8000	Glycerol
*k*_inactivation_^a^	0.441	0.088	0.103
C_1/2_^b^	1.6	7.8	6.7

^a^The rate constant of inactivation (*k*_inactivation_), expressed as 1/% (w/v) of the additive per reaction volume, was estimated by plotting the ln percentage residual laccase activity versus the percentage of additive in the reaction trial for laccase

^b^The C_1/2_, expressed in % (w/v) of the additive per reaction volume, defined as the additive concentration at which 50% of the initial activity was reached

#### Effects of selected additives on laccase thermostability

The effects of selected additives, including CuSO_4_, PEG-8000 and glycerol, on laccase activity at different incubation temperatures were investigated. Table 5 shows that the residual laccase activity of the control trial without additives was 81.0, 65.7, 49.0 and 4.1% after 168 h of incubation at 4, 25, 37 and 50 °C, respectively. A significant stabilization was obtained with the addition of CuSO_4_, where 116.4, 88.0, 62.8 and 12.6% of the laccase activity was retained after 168 h of incubation at 4, 25, 37 and 50 °C, respectively. The addition of 0.02% (w/v) PEG-8000 to the reaction mixture, resulted by 112.5, 96.2, 99.3 and 11.8% residual laccase activity after 168 h of incubation at 4, 25, 37 and 50 °C, respectively. In the presence of 0.4% (w/v) glycerol, the laccase activity was 114.1, 94.0, 79.2 and 9.8% of its initial activity, after 168 h of incubation at 4, 25, 37 and 50 °C, respectively.

**Table 5:** Effect of additives used in the reaction mixture on laccase thermal stability in aqueous medium composed of acetate buffer (0.1 M, pH 5.0), using syringaldazine as substrate

Additive	Relative concentration^a^	Residual laccase activity (%)^b^			
		4 °C	25 °C	37 °C	50 °C
None		81.0 (3.0)^c^	65.7 (4.2)	49.0 (7.3)	4.1 (0.7)
CuSO_4_	0.002	116.4 (1.6)	88.0 (5.1)	62.8 (9.6)	12.6 (4.6)
PEG-8000	0.02	112.5 (4.0)	96.2 (1.3)	99.3 (7.6)	11.8 (0.3)
Glycerol	0.4	114.1 (10.1)	94.0 (10.8)	79.2 (1.3)	9.8 (0.4)

^a^Relative concentration of the additive, expressed in percent (w/v), was the one at which the laccase specific activity was at its optimum

^b^Residual laccase activity (%) was calculated by dividing the specific activity (nmol product/mg protein/min) of a given sample after 168 h of incubation at a defined temperature to that at time 0, multiplied by 100. The enzyme activity was assayed with syringaldazine as substrate

^c^Relative percentage standard deviation was defined as the standard deviation of laccase triplicate trial divided by their respective means, multiplied by 100

The laccase thermal stability at 50 °C, was also investigated over 7 days period of incubation. The semi-logarithmic plots of the inactivation kinetics, fitted in linear regression curves and with high correlation coefficient values, indicate (Table 6) that the thermal inactivation of laccase at 50 °C followed the first order kinetic behavior. The results also show the rate constant of deactivation (*k*_t_) and half-life time (*t*_50_), estimated from the semi-logarithmic plots. The *t*_50_ for the laccase control trial at 50 °C was 38.9 h, whereas that in the presence of CuSO_4_, PEG-8000 or glycerol was of 52.9, 54.6 and 50.2 h, respectively.

**Table 6:** Effect of additives used in the reaction mixture on laccase thermal stability in aqueous medium composed of acetate buffer (0.1 M, pH 5.0), using syringaldazine as substrate

Thermal inactivation parameter	Additive			
	None	CuSO_4_ 0.002% (w/v)	PEG-8000 0.02% (w/v)	Glycerol 0.4% (w/v)
*k*_t_ (h^−1^)^a^	0.0178	0.0131	0.0127	0.0138
T_50_ (h)^b^	38.9	52.9	54.6	50.2

^a^Constant of inactivation was determined from the first order kinetics behavior of the inactivation effect of increasing incubation time at a specific temperature

^b^T_50_ is defined as the incubation time at the specific temperature in acetate buffer (0.1 M, pH 5.0) required to report a 50% decrease in the initial activity

## Discussion

The experimental findings suggest that the addition of mannitol, dextran, sucrose and the mixture BKSS enhanced the laccase activity of the enzymatic extract from *C. hirsutus* during freeze-drying as compared to the control without additives. Moreover, mannitol and dextran were the most appropriate lyoprotectants for the preparation of highly soluble powder freeze-dried laccase. Stepanova et al. (2003) showed that the addition of 1% (w/v) dextran 17 kDa resulted in 95 and 88% of residual laccase activity after a vacuum-drying of the enzymatic extract, obtained from *C. hirsutus* and *Coriolus zonatus,* respectively. Urena et al. (2016) reported that the use of the lyophilization agent maltodextrin in an aqueous suspension of the enzyme laccase from *Trametes versicolor* was an optimum mixture for the bio-functionalization of carbon surfaces. Hall et al. (2008) indicated that the residual activity of lipoxygenase (LOX) and hydroperoxide lyase (HPL) of the enzyme extract, obtained from *Penicillium camemberti*, was 55.0 and 29.7%, respectively, when 60% (w/w) dextran 72.2 kDa was added to the enzymatic extract before its lyophilization; these authors also reported that the addition of 86% (w/w) of KCl maintained 92.9% of the LOX residual activity and resulted by 2.25 times of enhancement of the HPL activity. In contrast, Capolongo et al. (2003) findings indicated that the addition of mannitol and dextran to the lignin peroxidase extract was not suitable to protect the enzymatic extract during its lyophilization, since their interactions with the protein destabilize it by decreasing its unfolding temperature; these authors also indicated that although the sucrose had a stabilizing effect on the lignin peroxidase, the sucrose containing-solutions were difficult to be lyophilized since the glass transition was at − 33 °C, which resulted in a rubbery product after their lyophilization. The stabilization of enzyme activity, obtained by the addition of sugar and poly-alcohol, could be related to their ability to replace the water molecule by involving the hydrogen bonding with polar groups on the protein (Crowe et al. 1993; Allison et al. 1998). Tanaka et al. (1991) demonstrated that sugars and poly-alcohols could protect the catalase by helping their direct interaction with the protein. The beneficial lyoprotectant effects of dextran and mannitol may be also attributed to the alteration of the glass transition temperature (*T*_*g*_) of the enzymatic preparation (Carpenter et al. 1997). Wang (2000) suggested that the *T*_*g*_ of the stabilized formulation is reached, the greater the degree of structural preservation is obtained with less protein aggregation.

The results reported in this study also suggested that the laccase stability is dependent on the dextran molecular weight. Similarly, Gloger et al. (2003) indicated that the addition of 4% (w/v) dextran 10 kDa provided the highest relative residual activity of 104% for the carbohydrate-binding activity of aviscumine, whereas the addition of the same concentration of dextran 75 and 1 kDa resulted by 83 and 92% of its residual activity, respectively. These authors also reported a 20% decrease in the protein-binding activity of aviscumine when the molecular weight of dextran was increased from 1–75 kDa. The inactivation effect obtained with the use of large dextran polymer (40 and 70 kDa), may be attributed to the ability of the dextran to change the glass transition temperature (*T*g) of a protein formulation (Wang 2000). High molecular weight dextrans show more intramolecular hydrogen bonds which lead to a loss in intermolecular bonding capacity resulting in decreased protein stability (Tanaka et al. 1991). The failure of dextran with very low or very high molecular weight to stabilize laccase activity may be due also to the low or high content in residual water in the lyophilized enzyme preparation resulting with under- or over-drying of the protein (Carpenter et al. 1997; Gloger et al. 2003). The reasons of the instability of laccase in presence 40 kDa dextran are not clear. This could be related to proteins impurities or to the presence of two laccase isozymes in the enzymatic extract obtained from *C. hirsutus* as characterized by sodium dodecyl sulfate polyacrylamide gel (SDS-PAGE) of the concentrated protein extract, the partially purified and purified enzymatic fraction, with estimated molecular weights of 31 and 56 kDa (Taqi 2012).

Moreover, the experimental findings suggested that the stabilization effect, obtained with the use of dextran or mannitol, was concentration dependent. Similarly, Hall et al. (2008) indicated that the increase in dextran concentration was associated with a decrease in LOX and HPL activities. On the other hand, Stepanova et al. (2003) reported that the increase in dextran concentration, from 0.5 to 1%, resulted by an increase in the residual laccase activity, from 67 to 95%, after a vacuum-drying of the enzyme extract; these authors also reported that the increase in the polyvinyl alcohol concentration, from 3 to 5%, resulted in a decrease from 75 to 69% of the enzyme activity, obtained from *C. hirsutus*, and an increase, from 48 to 85%, in that obtained from *C. zonatus*. It was demonstrated that the maximum protection of a protein by carbohydrates is achieved at the concentration that allow forming a monomolecular layer on the protein surface (Tanaka et al. 1991). It is also suggested that the optimal protection effect is obtained by amorphous mannitol, but the stabilizing effect decreases with an increase in its crystallinity (Izutsu et al. 1994).

Similar to the findings reported in this study, the increase in laccase activity of the enzymatic extract after a pre-incubation in the acetate buffer at different temperature and incubation time was also reported for other fungal laccases, including those from *Fomes sclerodermeus* (Papinutti et al. 2008), *Chaetomium thermophilum* (Chefetz et al. 1998) and *C. zonatus* (Koroleva et al. 2001). The activation of laccase after the thermal treatment could be due to a change either in the ratio of monomeric and aggregated enzyme molecules in solution or in the conformation of its active site (Stepanova et al. 2009).

In addition, the activation rate, obtained during the pre-incubation of the different laccase preparations at different temperatures, was relatively higher than that for the fresh enzyme preparation reported previously by Taqi (2012). Similar findings were reported by Stepanova et al. (2009) where the activity of the native laccase preparation, cooled to 4 °C, did not vary during the subsequent incubation for 4 h at room temperature, whereas that of the frozen one at − 18 °C did not exhibit any enzyme activity immediately after it was thawed. Yaropolov et al. (1994) suggested that the activation of laccase, obtained after thermal pre-incubation, could be attributed to the effect of such treatment on the transition of the enzyme state from the so-called dormant form to the active one. Stepanova et al. (2009) reported that the ratio of monomeric and aggregated laccase molecule was unchanged before and after incubation at room temperature, whereas the structure of all the three copper centers (Type-I ion copper, Type-II and Type-III) was rearranged during the incubation. On the other hand, Koroleva et al. (2001) reported that the decrease in laccase activity at the temperature range of 55–65 °C was caused by the release of Type-II ion copper which was completely absent at 70 °C; in addition, the Type-I and Type-III sites were completely disintegrated at temperatures higher than 70 °C, while the overall protein conformation was still maintained intact.

Moreover, the overall results show that the reconstituted laccase was stable at different thermal treatment, while the use of dextran and mannitol did not confer any significant stabilization of laccase as compared to the control trial. Papinutti et al. (2008) indicated that, with or without mannitol, the residual laccase activity of *F. sclerodermeus* after 24 h of incubation at pH 4.5 and at 40 °C was 50%. Moreover, Stepanova et al. (2009) reported that dextran did not provide an appropriate stabilization effect on the laccase activity of *C. hirsutus* and *C. zonatus*, after 196 h of incubation at 40 °C, with a residual enzymatic activity of 16.1 and 23.5%, respectively. Hall et al. (2008) reported that the addition of 5% (w/v) mannitol to the reaction mixture resulted by an increase in the thermostability of LOX from *P. camemberti*.

The experimental findings (Fig. 4) also suggest that the laccase preparation with mannitol and the control without lyoprotectant showed higher thermostability when it was stored for 1–4 h at 25 and 50 °C as compared to shorter incubation time. Similar findings were also reported by Saparrat et al. (2008) in which the relative laccase activity of the crude enzyme, obtained from *Grammothele subargentea*, increased after 4 h of its incubation at 40, 50 and 60 °C. The results are also in agreement with those of Chefetz et al. (1998) in which a preincubation of the purified laccase, obtained from *C. thermophilum*, at 40 to 60 °C and up to 1 h increase the enzyme activity. This increase could be due to conformational changes of the enzyme, which may increase its flexibility and therefore its catalytic activity. Saparrat et al. (2008) suggested that conformational changes in metalloproteins, such as laccase, can lead to more efficient electron transfer rates in the reaction. Coll et al. (1993) have reported that fungal laccases may be thermally activated by their pre-incubation at elevated temperatures up to 60 °C. In addition, it is suggested that mannitol did not have significant effect on the conformational changes of the enzyme in solution during thermal treatment.

The assessment of the storage stability of the different laccase preparation for 4 weeks at − 80, 4 and 25 °C showed that with mannitol was the most stable as compared to that with dextran and the control lacking any additive. Wang (2000) reported that the presence of additives in an enzyme preparation may have adverse effects, which could be due to the crystallization of the additives during storage; the destabilization can be also attributed to the failure of the dextran to interact effectively, by hydrogen bonding, with the protein molecules. The stabilization mechanism of the additives could be attributed mainly to their ability to inhibit the aggregation of the protein by physical or chemical interactions, the chemical degradations, the deamination, the oxidation of side chains or the hydrolysis of the lyophilized protein formulations.

The effect of additives and their concentrations on the laccase activity and its activation rate were also investigated. The results show that PEG-8000 was the most appropriate additive for laccase activity, followed by glycerol, while the addition of CuSO_4_ resulted by a decrease of the enzyme activity. Stepanova et al. (2003) reported that the addition of various salts (10^−3^–10^−1^ M) to laccase suspensions showed a concomitant decrease in the laccase activity with the increase in salt concentrations; these authors also reported that the addition of Cu^2+^ to the enzyme suspension resulted by a 25% decrease in its activity. Kim and Nicell (2006a) reported also that the laccase suspension, treated with 1 mM cyanide, Cu^2+^ and Fe^3+^, showed a significant decrease in the conversion of triclosan by 55.8, 28.0 and 6.2%, respectively; since this decrease may be due to the fact that these ions may interrupt the electron transport systems of the enzyme activity decreasing hence its activity. Modaressi et al. (2005) reported that the PEG-3350 was able to increase (20%) the laccase activity, with a turnover of fivefold toward triclosan. Kim and Nicell (2006b) reported that PEG-3350 had shown to be the most effective additive for the enhancement of the conversion of bisphenol A, by preventing the laccase inactivation rather than by increasing the reaction rate. Ghosh et al. (2008) suggested that the presence of PEG and glycerol in the laccase reaction solution may reduce the destabilizing effect of the products by hindering their hydrogen bonding site with the hydroxyl end group of those additives. It is also suggested that the presence of polyols in the laccase reaction media act as a water-structure maker which depresses the hydration of the enzyme and hence its denaturation (Papinutti et al. 2008). Although the metal ions including copper play crucial role as enhancers of laccase activity, especially that laccases have four copper atoms in their catalytic center, it could inhibit it at varying degrees, yet the destabilizing mechanism was not elucidated yet (Hernandez-Monjoraz et al. (2018).

The effects of selected additives, including CuSO_4_, PEG-8000 and glycerol, on laccase activity at different incubation temperatures were investigated. The overall results suggest that the laccase showed a higher residual activity in the presence of the investigated additives as compared to that of the control trial after 168 h of incubation at different temperatures. Poonkuzhali and Palvannan (2011) also reported that the addition of Guar Gum, Starch, agar and agarose to the laccase *Pleurotus florida* at different temperatures (50, 55, 60 and 65 °C) increased the enzymatic activity when compared to the control and the reported results demonstrated the ability of those additives in converting thermolabile laccase into a thermostable one attributed to the gelling nature of polysaccharide additives.

The laccase thermal stability at 50 °C was also investigated over 7 days period of incubation. The overall findings suggest that the investigated additives conferred a close level of stabilization for the laccase activity at 50 °C, by extending their half-life time values. Papinutti et al. (2008) reported that the highest stabilization effect was obtained when laccase from *F. sclerodermeus* was incubated in the presence CuSO_4_; these authors also reported that the combination of 1.25 mM CuSO_4_ and 0.2% glycerol conferred an increases in the half-life time value for laccase of 114, and 9.81 h as compared to the control, when incubated at 40 °C, respectively. Baldrian and Gabriel (2002) indicated that the stability of laccase, from *Pleurotus ostreatus*, was increased in the presence of Cu^2+^ with a residual enzymatic activity of 45% as compared to 27% of that of the control, after 7 days of at 20 °C.

On the other hand, Stepanova et al. (2003) reported on the effects of dextran, lactitol and polyacrylic acid, used as additives, on the thermal stability of laccase, from *C. hirsutus* and *C. zonatus*, incubated at 40 °C for 196 h, and indicated that neither the individual additives nor their combination had provided stabilization for both laccases. The literature (Koroleva et al. 2001; Papinutti et al. 2008) suggested that the thermal inactivation of laccase may be due to the depletion of the copper ion from the enzyme center, though the stabilization of laccase by the presence of CuSO_4_ could be due to the effect of the Cu^2+^ in delaying the release of the copper center from the laccase and preventing hence its inactivation. The mechanism of laccase stabilization conferred by PEG and glycerol might be related to their effects by decreasing the water activity or by regulating the interactions between the water molecule and the proteins (Kim and Nicell 2006a).

The experimental data obtained throughout this study showed that the optimal lyophilized laccase preparation was achieved with the use of mannitol as lyoprotectant. The lyophilized laccase was stable at various storage conditions and the reconstituted enzyme was stable at different incubation temperatures. The results also indicated that the addition of selected chemicals to the reaction medium resulted by an increase in the enzyme thermal stability. The enhanced stability of the laccase enzymatic extract could provide a better use of these enzymes in various biotechnological applications.

## Abbreviations

ATCC: American Type Culture Collection
BKSS: sodium benzoate, potassium sorbate and sorbitol
BSA: bovine serum albumin
HPL: hydroperoxide lyase
LOX: lipoxygenase
PEG: polyethylene glycol
RSD: relative standard deviation
SD: standard deviation
SG: syringaldazine
*T*_g_: glass transition temperature
